# Why Are Girls Less Physically Active than Boys? Findings from the LOOK Longitudinal Study

**DOI:** 10.1371/journal.pone.0150041

**Published:** 2016-03-09

**Authors:** Rohan M. Telford, Richard D. Telford, Lisa S. Olive, Thomas Cochrane, Rachel Davey

**Affiliations:** 1 Centre for Research and Action in Public Health, Health Research Institute, University of Canberra, Canberra, ACT, Australia; 2 Medical School, College of Medicine, Biology and Environment, Australian National University, Canberra, ACT, Australia; 3 Research Institute for Sport and Exercise, University of Canberra, Canberra, ACT, Australia; 4 Department of Psychology, Australian National University, Canberra, ACT, Australia; Vanderbilt University, UNITED STATES

## Abstract

**Background:**

A gender-based disparity in physical activity (PA) among youth, whereby girls are less active than boys is a persistent finding in the literature. A greater understanding of the mechanisms underlying this difference has potential to guide PA intervention strategies.

**Methods:**

Data were collected at age 8 and 12 years (276 boys, 279 girls) from 29 schools as part of the LOOK study. Multilevel linear models were fitted separately for boys and girls to examine effects of individual, family and environmental level correlates on pedometer measured PA. Cardio-respiratory fitness (multi-stage run), percent fat (DEXA), eye-hand coordination (throw and catch test) and perceived competence in physical education (questionnaire) were used as individual level correlates. At the family level, parent’s support and education (questionnaire) were used. School attended and extracurricular sport participation were included as environmental level correlates.

**Results:**

Girls were 19% less active than boys (9420 vs 11360 steps/day, p<0.001, 95%CI [1844, 2626]). Lower PA among girls was associated with weaker influences at the school and family levels and through lower participation in extracurricular sport. School attended explained some of the variation in boys PA (8.4%) but not girls. Girls compared to boys had less favourable individual attributes associated with PA at age 8 years, including 18% lower cardio-respiratory fitness (3.5 vs 4.2, p<0.001, CI [0.5,0.9]), 44% lower eye-hand coordination (11.0 vs 17.3, p<0.001, CI [5.1,9.0]), higher percent body fat (28% vs 23%, p<0.001, CI [3.5,5.7]) and 9% lower perceived competence in physical education (7.7 vs 8.4, p<0.001, CI [0.2,0.9]). Participation in extracurricular sport at either age 8 or 12 years was protective against declines in PA over time among boys but not girls.

**Conclusion:**

Girls PA was less favourably influenced by socio-ecological factors at the individual, family, school and environmental levels. These factors are potentially modifiable suggesting the gap in PA between boys and girls can be reduced. Strategies aiming to increase PA should be multicomponent and take into consideration that pathways to increasing PA are likely to differ among boys and girls.

## Introduction

A gender-based disparity in physical activity (PA) among youth, whereby girls are less physically active than boys, is a persistent finding in the literature [1–3]. While the magnitude of the difference in the amount of PA performed between boys and girls differs across studies, a large pooled investigation of European youth aged between 4–18 years indicates that girls perform on average around 17% less total daily physical activity [4]. Understanding the factors underlying gender differences in PA among youth has the potential to guide intervention strategies particularly in primary school-based settings, where boys and girls are most commonly taught physical education together.

Previous research points to several possible explanations as to why girls are less physically active than boys. Girls have been shown to participate less in organised sport [5], may receive less social support to engage in PA [6], and may perceive less enjoyment when taking part in physical education [7]. Recent evidence also suggests that relationships between physical and social environment correlates and PA may differ between boys and girls [8]. Biological reasons may also contribute to sex differences in PA. Differences in PA levels between boys and girls have been shown to be reduced after adjusting for sexual maturity [9], which suggests that lower PA levels in girls may be related to maturing at an earlier chronological age. Collectively, previous research points to a number of potential factors that underpin gender differences in PA but for the most part, these factors have been examined in isolation. This makes it difficult to understand the relative importance of each factor and to translate previous findings into practical intervention strategies.

Given the complexity of PA behaviour, with influences occurring at varying levels of the child’s experience, studies using a multilevel framework are needed to avoid analysing multilevel indicators in a single level framework [10]. An increasingly popular framework to examine variables of interest is the socio-ecological framework [11], which recognises multiple areas of influence on PA including the individual, family and environmental levels. A gap currently exists in the literature in examining variables at each of these levels concurrently to explain gender differences in PA, particularly using a longitudinal design. Whether influences on PA remain the same in boys and girls as they age is yet to be clearly established in the literature. It should not be assumed that the reasons behind gender differences are static or remain unchanged, particularly given that declining PA with increasing age may occur at different rates in boys and girls [12].

The aim of the current study was to investigate gender differences in PA using set of potential individual and contextual explanatory variables. Three domains were explored using variables previously shown to be correlated with PA in youth: 1) the Individual level: cardio-respiratory fitness [13], body composition [14], perceived competence in PE [7] and eye-hand coordination [15]; 2) the family level: parent’s support for physical activity [16] and level of parent education [17]; and 3) Environmental level: the influence of the school attended [18] and extracurricular sports club [19]. We extend previous research by, firstly, comparing explanatory variables of PA in boys and girls at age 8 years, then determining whether the explanatory variables are sustained through to age 12 years, and finally determining the effect of changes in these variables from age 8 to 12 years on changes in PA over time.

## Methods

This study is part of the ongoing multidisciplinary Lifestyle of our Kids (LOOK) project [20] which commenced in 2005. Participants from 30 government-funded schools, from outer suburbs of a city of population 325,000 were invited to participate through the school principals; 29 schools accepted. The number of schools was limited predominantly by our resources and commitment to schools to complete measures during a 10 week school term. All children enrolled in grade 2 from these schools were then invited to participate in the study of which 88% accepted (N = 853) by written consent provided by parents. The overall study incorporates measures of PA, fitness, motor control, psychological health, family influences, bone health, cardiovascular function, academic achievement and nutrition. This study involved data collected at age 8 years (baseline) and age 12 years.

### Physical activity, Fitness and Body composition

Physical activity was measured using pedometers (Walk 4 Life, Plainfield, IL, USA) over seven consecutive days. As previously suggested [21], daily step counts less than 1000 and greater than 30,000 were considered erroneous and discarded. In order to maximise data, all valid days of pedometer data were used in the analyses and incomplete data were adjusted, taking into account between child variation, sex, and day of the week differences using a Best Linear Unbiased Predictor as previously described [22]. This effectively removes the inherent skewness of the absolute number of steps per day by scaling PA with the square root. Cardio-respiratory fitness (CRF) was measured using the 20m multistage run, a well-established field test for children [23]. In each data collection phase, the same technician conducted both the fitness and PA measures. Body composition was measured using dual emission x-ray absorptiometry (DEXA, Hologic Discovery QDR Series, Hologic Inc., Bedford, MA, USA) and Hologic Software Version 12.4:7 was used to calculate percent body fat. Height (portable stadiometer to the nearest 0.001m), weight (portable electronic scales to the nearest 0.05 kg) and BMI (kg/m^2^) were measured in participants without shoes and wearing light clothing using standard measures.

### Pubertal development

At age 12 years pubertal development was determined by a self-assessment of Tanner stages using diagrams. The self-assessment occurred in a hospital setting with guidance from an experienced teacher. Children were classified as early-pubertal, mid-pubertal and late/post-pubertal if their breast/penis and pubic hair Tanner cumulative score was ≤4, 5–6 (and girls reported no menarche) and ≥7 (and/or they reported menarche), respectively.

### Eye-hand coordination

To provide an objective assessment of eye-hand coordination (EHC), a simple throw and catch skill test was developed [24]. Participants threw a tennis ball overhand against a solid unmarked wall and attempted to catch the rebound. There were two sets of 20 throw and rebound catch attempts at progressively increasing distances of 0.5, 1, 1.5, and 2 m from the wall. The children threw the ball with their preferred hand. In the first set, the children were asked to catch the ball with two hands and in the second set with one hand. Successful catches were recorded, the maximum score being 40, with higher scores indicating better EHC.

### Parent questionnaire

Questionnaires were completed by parents/carers as part of a more comprehensive questionnaire on family lifestyle and medical history. Responses from the survey were used to obtain measures of a) parents education level b) whether their child participated in organised sport and c) the level of support parents provided for their child to be physical active. Parent support for PA was determined from responses to the following four questions: In the last week how many times have you 1) Provided transport for your child to do physical activity or sport, 2) Watched your child being physically active or playing sport, 3) Talked about the benefits of doing physical activity, 4) Supported any other children to be physically active or play sport. A five point scale for the responses was applied to each item (1 = none, 2 = 2 to 3 days, 3 = 3 to 4 days, 4 = 4 to 5 days, 5 = daily). The total combined score was used to assess the level of parent’s support. This scale demonstrated acceptable internal reliability (Cronbach’s alpha = 0.69).

### Child questionnaire

As part of a more comprehensive questionnaire on physical activity and psychological health, perceived competence in PE was measured. Using a 5 point Likert scale (1 = Strongly disagree; 2 = Disagree; 3 = Not sure; 4 = Agree; and 5 = Strongly agree) participants were asked to respond to the following statements 1) I am good at PE and 2) My teacher thinks I am good at PE. This scale was shown to have sound internal reliability (Cronbach’s alpha >0.70 at both assessments).

### Statistical analysis

All analyses were conducted in R [25]. Firstly, independent samples t-test was used to compare the means of each explanatory variable of interest in boys and girls at age 8 and 12 years. Secondly, the *lmer* function from the *lme4* [26] package was used to perform a linear mixed effects analysis of the fixed effects for individual, family and environmental level variables on the outcome variable PA. Our data were structured such that participants were nested within schools. As this structure may result in non-independent data, whereby participants attending the same school may have a tendency to be similar in physical activity levels, a random intercept effect term for School was examined. A significant likelihood ratio test comparing the null multilevel model with a null school-level model justified the inclusion of School as a random intercept term in subsequent models. Variables (%BF, CRF, EHC, PE competence, level of parent education, parent’s support for PA and Sports club participation) were introduced to each model.

Separate models were created for girls and boys because of the known differences in physical activity levels, and plausible biological sex differences that could influence physical activity, including the timing and tempo of growth and maturation and differences in body composition. Models were fitted for boys and girls at age 8 and 12 years and stage of maturation was included in the analysis at age 12 years. The significance of each variable was assessed by fitting a series of models using backward-step elimination and comparing each model using a log likelihood ratio test. Where a variable under consideration did not significantly improve the model fit, that variable was removed, otherwise it was retained. In order to examine whether change in any of the examined variables explained change in PA across time, change scores, with the exception of sports club participation and stage of maturation, were calculated for each variable and the modelling procedure outlined above was repeated. For these models sports club participation which was coded as a binary variable 1 = participated in a club sport at either time point or 0 = did not participate in a club sport at either time point. In the absence of baseline pubertal assessments and based on the assumption that children were pre-pubertal at age 8 years, stage of maturation at age 12 years was entered into the models examining change in PA. To estimate the magnitude of the between school variance in PA the variance partition coefficient was calculated. Routine model checking procedures, including visual inspection of residual plots were used to check for deviations from homoscedasticity or normality.

The study was approved by the ACT Department of Education and Training (2013/00082-5), the Australian Institute of Sport Ethics Committee (2006/06/06) and the ACT Health Committee for Ethics in Human Research (ETH.9/05.697), and written informed consent was obtained from parents.

## Results

A summary of participant numbers and characteristics is shown in Table 1. Overall, 15 of the 853 children who provided consent to participate in the LOOK study withdrew and 146 children left our cohort because they relocated to a school outside the study area. Measurements were collected on different days and participant numbers were affected by absence from school on a day of testing. At baseline, 372 boys and 366 girls returned valid pedometer data from which a total of 276 boys and 279 girls also completed other assessments of interest for the present investigation and were included in the analyses. Of these participants, 175 boys and 186 girls completed assessments of interest at follow-up. There were no significant differences at baseline for height, weight, %BF, CRF, or SES between those who completed all measures and were included in the analyses, in comparison to those who did not and were excluded. However, of interest but of no consequence to our gender comparisons, baseline level of parent’s support was 13 percent higher for children who completed follow-up measures (M = 14.06, SD = 3.80) compared to those who did not (M = 12.44, SD = 4.42; t = 2.97, p = 0.003).

**Table 1:** Participant Characteristics and significance test for differences in mean characteristics between boys and girls at age 8 and 12 years.

	Age 8 years					Age 12 years				
	Girls N = 279		Boys N = 276			Girls N = 186		Boys N = 175		
	Mean	sd	Mean	sd	P	Mean	sd	Mean	sd	P
Height (cm)	128.84	5.49	130.2	5.51	.008	154.03	6.91	153.56	7.72	.571
Weight (kg)	28.73	5.79	28.94	5.31	.664	46.88	10.33	46.34	9.77	.572
BMI	17.19	2.57	17.03	2.28	.557	19.74	3.43	19.50	3.20	.624
Percent body fat (%)	28.01	6.35	22.73	5.91	<.001	27.69	6.38	24.49	7.24	<.001
Cardio-respiratory fitness (stages completed)	3.53	1.08	4.18	1.48	<.001	5.61	1.83	6.44	2.12	<.001
Physical activity (steps/day)	9900	1702	12256	1876	<.001	8940	2611	10463	3423	<.001
Eye-hand coordination (number of catches)	11.04	9.41	17.30	12.72	<.001	31.72	7.41	35.72	5.66	<.001
Parent support	13.72	3.81	13.79	4.17	.822	12.88	3.51	14.12	4.04	.026
Physical Education Competence	7.73	2.10	8.37	2.04	<.001	7.62	1.70	8.51	1.71	<.001
Sports Club members (%)	67%		80%			69%		84%		
% meeting step/day recommendations**	37%		43%			30%		30%		
Parent level of education										
Year 10 or below	10%		14%			…		…		
Year 12	25%		24%			…		…		
Trade or higher education	65%		60%			…		…		
Puberty Status										
Early maturation	…		…			28%		44%		
Mid-maturation	…		…			60%		45%		
Late-maturation	…		…			12%		11%		

**Physical activity recommendations based on boys >13000 and girls >11000 steps/day [39].

### Characteristic differences between boys and girls

As shown in Table 1, at age 8 years during pre-pubescence, girls had higher levels of %BF (28% vs 23% p<0.001, 95% CI [3.5, 5.7]) than boys, despite no observed sex differences in weight, BMI or in BMI cut-off points [27] for normal, overweight and obese categories (boys; 79%, 16%, 5% vs girls; 74%, 18%, 8%, p = 0.14). Boys were on average 1.3 cm taller than girls at age 8years (p = 0.008). Girls also accumulated significantly less steps per day than boys (9900 vs 12256, p <0.001, 95% CI [1844, 2626), and had 18% lower CRF (3.5 vs 4.2, p<0.001, 95% CI [0.5, 0.9]) and 44% lower EHC scores (11.0 vs 17.3, (p<0.001, 95% CI [5.1, 9.0]). Perceived competence in PE was observed to be 9% lower among girls (7.7 vs 8.4, p<0.001, 95% CI [0.2, 0.9]) and a lower percentage of girls (60%) reported participating in an extracurricular sports club compared with 80% of boys (p<0.001). Although the magnitudes in these characteristics varied slightly, differences at age 8 years were again evident at 12years except for the reported level of parent’s support. Parents support for their child to be physically active was higher among boys compared to girls at age 12 years (12.9 vs 14.1, p = 0.026, 95% CI [-0.7, 0.8]), however, there was no significant difference at age 8 years. During the course of this 4 year study, there was a reduction in average PA from age 8 to 12 years among boys (-14%) and girls (-10%), but there were increases in CRF, EHC, height and weight in both sexes (Table 1). At age 12 years, a greater proportion of girls reported being in the mid-maturation stage of pubertal development compared to boys.

### Variables explaining physical activity

As shown in Table 2, multilevel models fitted separately for 8 year-old boys and girls revealed several common significant explanatory variables. PA was higher in boys and girls who participated in extracurricular sport (both p<0.001), had higher CRF (both p< 0.001) and superior EHC (both p<0.05); as was PA in girls with lower %BF (p<0.001). Our modelling also revealed that among boys, but not girls, higher perceived competence in PE (p<0.001) and higher levels of parent’s support (p<0.03) were associated with higher PA.

**Table 2:** Multilevel linear mixed effect models for boys and girls), examining the effects of individual, family and environmental level variables on physical activity and change in each variable on change in physical activity from age 8 to 12 years.

	Age 8 years						Age 12 years						Change from 8 to 12 years*					
	Boys (N = 276)			Girls (N = 279)			Boys (N = 175)			Girls (N = 186)			Boys (N = 175)			Girls (N = 186)		
	Estimate (SE)		p	Estimate (SE)		p	Estimate (SE)		p	Estimate (SE)		p	Estimate (SE)		p	Estimate (SE)		p
Physical Activity (Intercept)	89.31	6.21		88.08	6.09		61.57	9.52		62.01	8.22		-10.14	4.42		0.12	5.21	
***Individual Level***																		
Percent body fat	0.01	0.13	.08	-0.13	0.10	<.001	0.25	0.15	.120	0.31	0.15	.13	-0.44	0.25	.14	-0.01	2.09	.84
Cardio-respiratory fitness	1.68	0.52	<.001	2.57	0.69	<.001	1.94	0.58	<.001	3.50	0.62	<.001	0.36	0.48	.42	0.86	0.69	.10
Eye-hand coordination	0.11	0.06	.01	0.16	0.06	.02	0.44	0.24	.040	…	…		0.18	0.09	.08	…	…	
PE Competence	1.05	0.34	<.001	-0.16	0.31	.62	0.76	0.64	.200	0.87	0.55	.10	0.10	0.42	.57	-0.68	0.39	.08
Stage of Maturation**							-0.29	0.49	.54	-0.53	0.53	.31	-0.94	0.51	.07	-0.68	0.63	.28
***Family level***																		
Level of Education	-0.21	0.94	.79	1.19	0.91	.20	-1.11	1.11	.31	0.21	1.34	.97	-1.46	1.06	.32	-0.88	0.40	.48
Parental support of PA	0.34	0.17	.03	0.06	0.16	.56	…	…		…	…		…	…		0.02	1.34	.99
***Environmental level***																		
Sport club participation	1.48	1.67	<.001	2.80	1.31	<.001	5.22	2.74	<.001	-2.67	2.10	.25	5.65	2.11	.04	-1.43	5.17	.62
School level variance***	8.43	2.95	<.001	0	0		7.16	2.67	<.001	0	0		2.66	1.63	.20	0	0	

^…^ Term excluded from the final model.

* Change scores were calculated for each variable except for stage of maturation and sport club participation. Sport club participation was entered into the model as a binary variable 1 = participated in sport at either age 8 or 12 years, or 0 = did not participate in sport.

** Stage of maturation was not measured at age 8 years. Stage of maturation at age 12 was entered into the models examining change in physical activity.

*** School level variation in PA was estimated by variance partition coefficient.

PA was explained by fewer variables at age 12 years than at age 8 years. In particular, as shown in Table 2, the significant association between extracurricular sport and PA observed at age 8 years in girls was found to be non-significant at age 12 years. Table 2 also shows that CRF was the only significant explanatory variable in the fitted model for girls at 12 years (p<0.001). For boys, CRF (p<0.001), EHC (p = 0.02) and sports club participation (p = 0.001) remained significant explanatory variables for PA.

When investigating effects at the school level, there was evidence for a school effect on PA at both age 8 years and 12 years among boys but not girls, whereby the school attended by a boy accounted for 8.4% of the variation in PA scores at age 8 years and 7.2% at age 12 years (Table 2).

### Variables explaining change in physical activity over time

As summarized in Table 2, between 8 to 12 years, analyses in the boys showed that participation in an extracurricular sports club (p = 0.04) and trends towards improvements in EHC (p = 0.08), and later onset of pubertal development (p = 0.07) contributed to explaining change in habitual PA. In girls, none of the change variables contributed significantly to explaining changes in PA over time, although change in CRF (p = 0.10) and change in perceived competence in PE (p = 0.08) among girls were close to the significance threshold.

## Discussion

To address a gap in the literature in understanding why girls are less physically active than boys we employed a multilevel cross-sectional and longitudinal approach at the individual, family, and environmental levels of the socio-ecological framework. Our findings suggest that influences on PA at the school and family levels and through extracurricular sport participation are weaker in girls than boys. Moreover, girls were observed to have less favourable individual attributes associated with PA, including lower CRF and EHC, higher %BF and lower levels of perceived competence in PE.

One particularly interesting finding was that the school attended explained variation in PA levels of boys but not girls. While school level variation in PA has previously been reported [28–30], the present study highlights a gender difference in the influence of the school on PA. A previous study of 856 eleven year-old Canadian children found that the school accounted for 6.7% of the variation in objectively measured light to vigorous PA [28], comparable with our study, where 8.4% of school related variance was seen for boys. Currently, it is unclear exactly what school factors are most influential on PA, although there is some evidence to suggest that schools with formalised policies regarding PA have more active students [28]. One study in 92 British schools, which also found a significant school effect on PA, was not able to pinpoint factors of influence within the school, despite investigating several correlates of PA including school size, density location, availability of sporting facilities and play area [30]. This finding prompted the authors to suggest that psycho-social factors are more likely to contribute to school differences than the physical school environment. Although the physical aspects of each school were not examined, this may also be the case in the present study where government funded schools receive similar funding and resources.

Given that schools are often viewed as the ideal setting to promote PA [31], the lack of influence of the school on girls’ PA in the current cohort is concerning. In the context of previous research indicating that girls and boys may behave differently during school lunch breaks [32] and physical education [7], a plausible explanation is that some schools provide more opportunities for students to be physically active during these times but they are more readily accessed or desirable to boys. Future research should continue to focus on identifying modifiable social and physical school characteristics likely to promote higher PA, bearing in mind that these factors may differ among boys and girls.

This study emphasises the central role of extracurricular sport as a contributor to PA among youth. A positive association of sports club participation with PA observed in both boys and girls has previously been reported in this cohort [33] and elsewhere [34], supporting the need for strategies to increase and maintain sport participation rates. Such strategies may be particularly relevant in girls, given our evidence that sports club exerts a diminished influence on PA as they approach adolescence. For boys, the reverse is true, where longitudinal participation in organised sport from age 8 to 12 years provided a protective effect against a decline in PA. In conjunction with our finding that PA levels vary between schools, our data provide support for a previously suggested strategy in which schools and sporting organisations and clubs work together to increase the skills, knowledge and motivation required for sustained participation in organised sport [35].

Given that boys and girls commonly participate in physical education together (at least between ages 8 and 12 years), it is interesting to consider gender differences in individual level characteristics (%BF, CRF, EHC), each of which have been shown to be associated with PA among youth [24,36,37]. For example, during pre-pubescence an 8 year-old girl of average weight (28kg) in comparison to a boy of equivalent average weight, will carry 2kg more body fat (and therefore approximately 2kg less lean muscle mass) and will already have poorer eye-hand coordination and fitness compared to boys. These differences alone illustrate that teachers, parents and coaches need to consider gender differences in mixed physical education and sport settings because activities that focus on physical performance are likely to favour boys, even before the onset of puberty. Teachers, in particular, need to know how to conduct PE and sport that provides boys and girls with equal opportunities for sustained engagement, development of competency and enjoyment of PA. This may be particularly relevant in primary school-based settings where classroom teachers, often with little background in PE, are largely responsible for physically educating boys and girls within an increasingly demanding school curriculum and administrative load.

The findings from the present study raise a key question that has implications for PA activity interventions. As a society, do we accept the premise that young girls are less physically active than boys as “normal” or is it because we are failing to provide girls with the same level of opportunity and support to be equally active? Our data cannot determine the answer with precision but are suggestive of the latter. For example, in the community setting, lower sport participation rates in girls may be indicative of fewer opportunities and/or less support for girls. Similarly, in the school setting, our data indicate that girls feel less competent in PE than boys, and that in contrast to boys, school had little influence on girls’ PA. Finally, the same trend is apparent in the family setting, with our data indicating that higher levels of parental support of PA is translated to higher levels of PA in boys but not girls. Given that each of these influences are potentially modifiable, it is possible that with increased support for girls’ PA at the school and family levels, these gender-related differences in PA during childhood could be reduced.

In this cohort the majority of boys and girls were insufficiently active according to recommended PA guidelines [38] and therefore strategies need to be put in place to increase PA. The complexity of PA behaviour suggests that interventions which operate on multiple levels of the socio-ecological framework are likely to have the best results. Our findings of fewer influences on PA at age 12 years compared to 8 years also indicate that interventions should be introduced at the youngest age possible and need to carefully consider equality of support and opportunities for girls and boys and how these needs change over time.

A strength of this study was the longitudinal design and multilevel framework which facilitated the examination of contributions of different levels of the socio-ecological framework on PA levels [10]. A further strength was the use of objective measures of body composition and PA. On the other hand, pedometers have a number of limitations in that they do not provide contextual information such as the type, intensity and duration of PA being performed. A further limitation of our study was that the examined variables are by no means exhaustive and other potentially important correlates of PA were not included. For example, this study did not capture neighbourhood environmental variables, which are known to influence PA. Another limitation was that participants in the current study were mostly Caucasian and from a jurisdiction of slightly higher SES than the Australian average, which should be taken into consideration when generalising our findings to other populations.

## Conclusion

In a population of children of mid-range socioeconomic status in Australia, lower PA among girls in comparison to boys can be explained, in part, by weaker influences on PA at school, through parent’s support and through lower participation in community sport. Because these influences are potentially modifiable, future intervention strategies to increase PA should focus on each of these areas simultaneously, and pay particular attention to equality of support and opportunities for girls and boys.
